# Biodegradable Microneedle for Enhanced Transdermal Drug Delivery: Trends and Techniques

**DOI:** 10.3390/mps8060134

**Published:** 2025-11-04

**Authors:** Renuka Khatik, Jatin Kumar Sahu, Shuvadip Bhowmik, Isha Rai, Madhu Kumari, Monika Dwivedi

**Affiliations:** 1 Mallinckrodt Institute of Radiology, Department of Neurology, Washington University School of Medicine, St. Louis, MO 63110, USA; khatik@wustl.edu; 2 Department of Pharmaceutical Sciences and Technology, Birla Institute of Technology, Mesra, Ranchi 835215, India; sahujatinkumar8@gmail.com (J.K.S.); shuvadipbhowmik8@gmail.com (S.B.); isharai2110@gmail.com (I.R.); madhukri505@gmail.com (M.K.)

**Keywords:** Transdermal Drug Delivery System (TDDS), biodegradable microneedles, biomacromolecule delivery

## Abstract

The Transdermal Drug Delivery System (TDDS) offers several benefits, such as enhanced patient adherence, controlled release, reduced gastric irritation, and the bypassing of the first-pass metabolism. However, not all drugs can be delivered through this route in effective doses. Biodegradable microneedles (BMn) are designed to improve TDDS. This review outlines various types of BMn and their fabrication methods. BMn are produced in different forms, including hollow, solid, dissolve, and hydrogel-forming versions, which have garnered significant attention. These innovative BMn do not contain drugs themselves but instead absorb interstitial fluid to create continuous channels between the dermal microcirculation and a drug-containing patch. Several types of BMn have been tested and approved by regulatory bodies. The use of BMn technology is rapidly growing in point-of-care applications, attracting significant interest from both researchers and healthcare providers. BMn-based Point-of-care (POC) devices have high efficacy for finding various analytes of clinical interests and transdermal drug administration in a minimally invasive manner owing to BMn’ micro-size sharp tips and ease of use. Porous BMn technology may have a very rising future in the case of a vaccine delivery system.

## 1. Introduction

Hypodermic needles are widely used in clinical practices to deliver medications through the skin and directly into the bloodstream. Although essential from a medical standpoint, these injections can be painful and may lead to side effects such as hypersensitivity, bruising, discomfort, and bleeding at the injection site. They also carry risks of contamination and accidental needle-stick injuries, necessitating proper training for healthcare professionals in their safe use. The primary barrier to transdermal drug delivery is the stratum corneum (SC), the outermost layer of the skin [1].

To address this, biodegradable microneedles (BMn) have emerged as an effective alternative, enhancing the delivery of both small and large molecules through the skin. Over the past few decades, advances in microfabrication technology, which are driven by academic research and pharmaceutical innovation, have enabled the precise development of these devices. BMn are engineered with micron-sized projections that create transient microchannels in the skin, allowing for efficient drug transport while minimizing pain by avoiding nerve stimulation. As a result, they improve patient comfort and compliance, particularly among individuals with needle phobia, since they are painless and suitable for self-administration [2].

**Figure 1:**
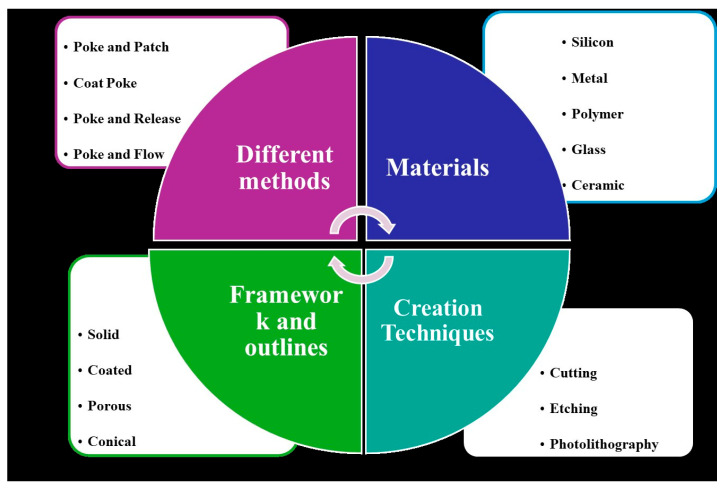
A schematic representation of BMn commonly utilized in biomedical diagnostics and therapeutic applications (created by the authors).

BMn are broadly classified into four main types: hollow, solid, coated, and polymer-based, as shown in Figure 1. Hollow BMn resemble conventional hypodermic needles but are shorter, delivering liquid drug formulations through internal bores. Solid BMn are used to create microchannels in the skin before applying a drug patch, while coated BMn are directly covered with the drug on their surface for immediate release upon insertion. Polymer-based BMn may be dissolving, hydrogel-forming, or non-dissolving, allowing for controlled and sustained drug delivery. These BMn arrays are virtually painless, pose a minimal risk of infection, with no reported cases to date, and can be self-administered.

However, several limitations persist. Silicon-based BMn may cause skin irritation since silicon is not FDA-approved as a biomaterial, and if a silicon or metal microneedle breaks within the skin, it could result in localized complications. Manufacturing costs remain high due to cleanroom requirements, and solid, non-coated BMn involve a two-step application process. Furthermore, achieving a uniform coating is challenging, limiting the dosage to small bolus amounts. Hollow BMn may also cause dermal compression or blockage, while heat exposure during polymer molding can degrade the drug. Additionally, studies have shown that microchannels created by BMn tend to close within two hours if not covered with occlusive dressing, which can restrict sustained drug delivery.

## 2. History of BMn

Hypodermic needles are common clinical devices used to draw or inject fluids or gases by puncturing the skin. They are among the most widely used devices globally, with applications in subcutaneous, intramuscular, and intravenous injections, blood collection, and various puncture techniques. However, their use is often associated with pain or discomfort. The limited absorption capacity of subcutaneous tissue and the size and properties of drug molecules have posed challenges for delivering certain medications. As a result, traditional transdermal drug delivery systems (TDDS), like patches, have remained limited in their application [3].

To address these limitations, advancements such as BMn have emerged. Gerstel and Martin from Alza Corporation first proposed the concept of BMn in 1976 as a solution to the pain associated with drug administration. This innovative idea laid the groundwork for microneedle development, though significant progress was not achieved until the 1990s, when advances in microfabrication techniques enabled the creation of BMn with precise designs and improved functionality. In 1998, a groundbreaking approach to transdermal drug delivery was introduced, significantly enhancing the transport of molecules across the stratum corneum [4]. BMn fabricated by etching arrays of microsized needles into silicon demonstrated a remarkable 1000-fold increase in skin permeability for certain molecules. This advancement spurred significant interest in the potential of BMn, inspiring further research into their capabilities [2].

A few years later, Mariano et al. reported the development of a commercial microneedle patch coated with ovalbumin (OVA) in 1 mg and 5 mg doses. These patches elicited immune responses up to 50 times greater than those achieved with equivalent doses delivered via traditional subcutaneous or intramuscular injections. This finding underscored the potential of BMn in enhancing immune responses, which are critical in the treatment and prevention of various diseases. Consequently, the development of microneedle patches has gained significant attention, with ongoing research exploring their applications as innovative and effective drug delivery systems [5]. In 2004, a BMn array was employed to create micro-perforations in the skin, enabling transdermal drug delivery [6]. This breakthrough spurred the exploration of numerous fabrication techniques and materials tailored for transdermal applications.

**Figure 2:**
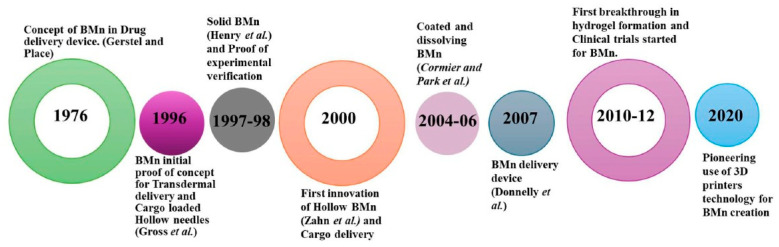
An overview of the advantages and disadvantages, fabrication techniques, and manufacturing limitations of BMn [11].

Additionally, various fabrication methods such as laser ablation, photolithography, and micro-injection molding have been explored for microneedle production. These technological advancements led to the first reported use of dissolvable microneedles for transdermal drug delivery in 2005, as illustrated in Figure 2 [7]. To date, over 40 clinical trials involving biodegradable microneedles (BMn) have been successfully completed, with the earliest trial concluding in 2007. More recently, additive manufacturing techniques have been employed to create BMn molds, offering a cost-effective and efficient approach to micro-mold fabrication [8,9]. In 2020, studies demonstrated the feasibility of using commercially available 3D printers to develop BMn master molds, representing a significant step forward in device design and enabling customized, scalable production of BMn [10].

## 3. Mechanism of BMn

BMn consists of two main components: the invasive section and the supporting structure. The invasive section is an array of numerous needles, typically ranging in length from 25 to 2000 microns. The supporting structure is a base plate designed to provide uniform mechanical support, ensuring the sharp needle tips can effectively penetrate the stratum corneum (SC). These two components can be fabricated from the same material or constructed separately using different raw materials before being securely bonded together [12]. The primary purposes of a microneedle patch (MNP) are to create micron-sized channels in the skin and to act as a drug delivery system. The effectiveness of an MNP depends on its design and requires sufficient hardness, appropriate mechanical strength, and adequate durability. Failures in an MNP are often indicated by issues such as needle tip bending, deformation, or breakage. These stringent requirements make the selection of suitable materials and the type of MNP design critical to its success [2].

**BMn-based transdermal delivery strategies:** The effectiveness of microneedle patch (MNP)–mediated drug-delivery largely depends on several key factors. These include the design characteristics of the MNP (such as structure, dimension, geometry, and the materials and fabrication methods used) as well as the nature of the therapeutic agent being administered. The different process can be classified as: “poke and patch”, “poke and flow”, “coat and poke”, and “poke and release” [13].

**Figure 3:**
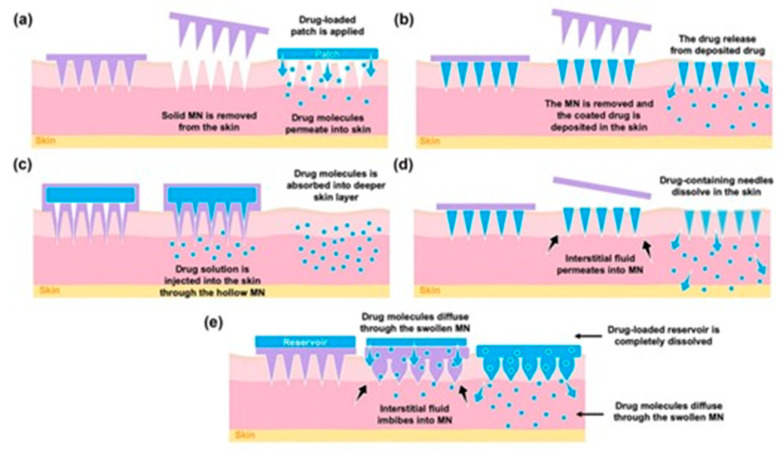
Drug delivery strategies using different types of BMn: (**a**) solid, (**b**) coated, (**c**) hollow, (**d**) dissolving, and (**e**) hydrogel-forming BMn. Reproduced with permission [20].

**Solid BMn for “Poke and Patch”:** The “poke and patch” technique utilizes solid microneedle arrays (MNAs) to puncture the skin, creating microchannels that reach the deeper layers of the epidermis [14]. This method significantly enhances passive drug transport through the skin by disrupting the stratum corneum, which serves as the primary barrier to permeability (Figure 3). The process involves two steps: first, the MNAs is used to create micropores in the epidermis and is then removed; second, the drug is applied in a conventional dosage form, such as a solution, cream, or patch, which acts as an external drug reservoir. This simplicity, especially from a mechanical standpoint, makes the technique highly appealing for clinical applications.

However, the approach is not without limitations. One significant drawback is that the micropores remain open for only a short duration, potentially leading to premature cessation of drug delivery. Studies have shown that microneedle-treated sites typically recover their barrier properties within two hours. Nonetheless, this timeframe can be extended to up to three days under occlusive conditions using formulations like patches or tapes. However, prolonged occlusion increases the risk of infection, which remains a critical consideration in this method [5,15]. For instance, Tsuchiya et al. [16] investigated the fabrication of a metal-based solid BMn using titanium. Another metal utilized for manufacturing solid BMn is stainless steel. Verbaan et al. [17] employed 30G stainless steel hypodermic needles as BMn material by cutting and screwing them to a poly(etheretherketone) mold. Ceramic-based BMn have been reported, with Bystrova and Luttge et al. [18] developing BMn by casting alumina slurry onto micromolds.

Different ceramic materials, such as calcium sulfate dihydrate, have been employed in the production of solid microneedles (SMNs). The release behavior of the drug and the mechanical durability were found to be influenced by the porosity and microstructure of the needles, as confirmed through scanning electron microscope (SEM) and microCT analyses. According to as described by Cai et al. [19] bioceramic BMn were developed that exhibit strong mechanical properties and are biodegradable, thereby supporting safe, and reliable transdermal drug delivery system (TDDS).

**Coated BMn for “Coat and Poke”:** This technique enables drug diffusion from the coated surface of Biodegradable microneedle arrays (BMNAs) into the deeper epidermal layers after insertion. However, several challenges limit the practicality of this approach. For instance, the amount of drug that can be encapsulated in the coating layer is relatively low [21]. Additionally, the thickness of the coating can reduce the sharpness of the BMn, affecting their ability to effectively penetrate the skin. Despite these limitations, coated BMNAs have demonstrated excellent efficiency in vaccination. This is because the antigen dose required to trigger an immune response is typically in the nanogram or microgram range, making the technique well-suited for such applications [22].

Lee et al. developed bleomycin-coated BMn designed to deliver bleomycin into the subepidermal derma layer, offering an effective and painless treatment for warts while minimizing patient discomfort and anxiety [23]. Tekko et al. (2020) [24] reported that Methotrexate sodium salt used as a main cargo commonly used for psoriasis treatment, but this salt is poorly hydrophilic and unsuitable for topical application. To address this, the drug was converted into nanocrystals and incorporated into dissolvable BMn. Upon skin insertion, the MTX nanocrystal-loaded BMn demonstrated efficient drug delivery, with approximately more than 300 folds higher skin accumulation compared to control (free MTX) in 24 h post-administration. In vivo studies in rats showed that 3 days after administration, around 12.5% of the MTX nanocrystals remained in the skin, indicating localized and sustained drug delivery achieved through the combination of dissolvable BMn and good nanomedicines [24].

**Dissolving and Hydrogel-Forming BMn for “Poke and Release”:** Dissolving biodegradable microneedle arrays (BMNAs) are fabricated from water-soluble and biodegradable materials that can encapsulate drugs for delivery. Upon insertion, the BMNAs dissolve, releasing the drugs directly into the skin. Compared to the “poke and release” method, dissolving BMn offers the advantage of maintaining controlled drug release over an extended period, achieved by adjusting the dissolution rate of the material used in the microneedle matrix. Another notable benefit is that this approach simplifies the drug administration process to a single step, as the BMNA punctures the skin and remains embedded until it fully dissolves [13]. Additionally, dissolving BMNAs eliminate the generation of sharp waste, reducing waste management costs and the risk of needle-stick injuries. However, the technique has some limitations, including lower drug-loading capacity and potentially reduced ability to penetrate the stratum corneum effectively [22].

Hydrogel-forming BMn have been utilized to enhance the transdermal absorption of various key compounds. Kearney et al. [10] demonstrated the effectiveness of hydrogel-forming BMn in improving the skin absorption of small molecules. In their study, hydrogel-forming BMn were paired with film reservoirs to deliver donepezil in a rodent model. Additionally, Donnelly et al. [25] explored the use of hydrogel-forming BMn combined with lyophilized wafers for delivering both small and large molecules, such as ibuprofen sodium and ovalbumin, respectively.

**Hollow BMn for “Poke and Flow”:** In this technique, BMn function similarly to hypodermic needles by enabling drug delivery after creating perforations in the skin [26]. However, their micrometric size makes the manufacturing process complex and costly, demanding advanced technological resources. Despite these challenges, the shorter size of these needles significantly improves patient acceptance compared to traditional injections, making this method more appealing for routine clinical use [27]. The application potential of hollow BMn was strongly validated when researchers observed that hollow BMn array delivery elicited a significantly stronger immune response compared to the intramuscular injection technique. Niu et al. further demonstrated that intradermal delivery using a hollow BMn designs a sustained-release pattern in the transdermal site while enabling a rapid to burst release via draining lymph nodes [28]. Hollow BMn are recommended for sample extraction due to their unique design and structure.

**BMn patches:** Micron-sized BMn penetrate the stratum corneum—the primary barrier to drug diffusion—creating pathways that allow medication to rapidly diffuse directly beneath the skin. This makes BMn effective drug delivery devices for percutaneous administration, enabling localized and systemic pharmacological effect [29]. In addition to their painless application, BMn have been used to enhance the bioavailability of protein-based drugs. They have made it possible to deliver complex molecules, such as proteins, vaccines, and peptides, via the transdermal route. BMn come in various types, including solid, hollow, coated, and dissolving designs, all aimed at achieving rapid and efficient drug delivery.

**Figure 4:**
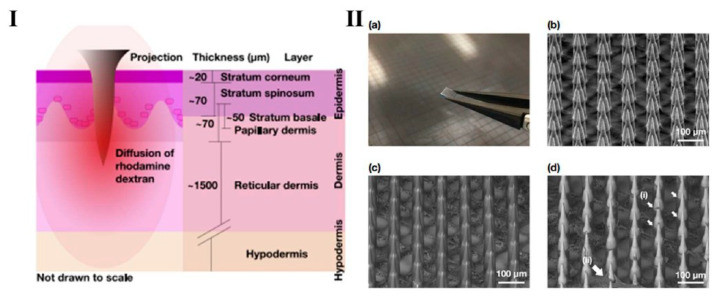
Graphic representation (**I**) Nano-patch micro projection employed to the skin, illustrating the diffusion of rhodamine dextran through the various layers of human skin. (**II**) (**a**) A photograph of a Nano-patch held with tweezers alongside a Nano-patch wafer; (**b**) A representative secondary electron detector SEM image of uncoated Nano-patch projections at a 45° tilt; (**c**) A representative backscatter electron (BSE) detector SEM image of a coated Nano-patch; (**d**) A representative BSE detector SEM image of a coated Nano-patch applied to full-thickness ex vivo human skin after removal. Reprint permission [32].

Jonathan, C.J. et al. studied the diffusion properties of macromolecules across each layer of human skin (illustrated in Figure 4I) following the application of a Nano-patch, an ultra-high-density BMn for vaccine delivery. Using rhodamine dextran with three different molecular weights relevant to vaccine and therapeutic applications, molecules were deposited at various depths within ex vivo human skin using the Nano-patch device (Figure 4II). The resistance to diffusion is primarily due to the strong cohesion of stratum basale (SB) cells, reinforced by compact junctions, desmosomes, and associate proteins [30].

In addition, the densely organized collagen invaginations within the papillary dermis further contribute to this structure, collectively forming an effective physical barrier against the penetration of large particles [31]. Furthermore, BMn systems offer controlled drug release and safer administration compared to surgical implantation methods, making them a versatile and innovative approach in modern drug delivery [5].

## 4. Materials Used for BMn

Various materials, ranging from metals to polymers, are used in BMn depending on the design and specific components of the device. In general, microneedle materials must possess sufficient mechanical strength to effectively penetrate the skin. Non-dissolving BMn are inert, biocompatible, and strong enough to pierce the skin without causing an immune response [33]. On the other hand, the materials used for coated and dissolving BMn are typically water-soluble and biocompatible. These materials are designed to dissolve or degrade in the body without inducing toxicity [22].

**Silicon:** Silicon offers sufficient mechanical strength for skin penetration, making it a commonly used material for the production of solid and coated BMn [14]. Silicon BMn can be precisely fabricated with small, sharp tips, typically less than 100 μm in length, using techniques such as deep reactive ion etching and photolithography [4]. However, the equipment required for these processes is expensive, the production is costly, and the manufacturing speed is relatively slow [34]. Additionally, silicon BMn may pose safety risks if they break off the skin, leaving fragments embedded in the tissue [35]. Recently, silicon has been increasingly used to create mold rather than in solid BMn [36].

**Metal:** Metal materials exhibit high mechanical strength and rigidity, allowing them to easily penetrate the skin. These properties make metals ideal for creating solid, coated, and hollow BMn. Stainless steel [13] and titanium (Ti) [21] are the most commonly used metals in BMn production. Stainless steel is the most frequently used metal due to its accessibility and affordability, but it corrodes more quickly than titanium alloys. Titanium alloys, while more expensive, offer superior mechanical strength compared to stainless steel [37]. While many metals are undoubtedly cheaper and stronger than silicon, there are concerns about the immuno-inflammatory response of soft tissue around stainless steel and titanium implants. Verbaan et. al. reported that the simplest form of solid metal BMs was made by assembling stainless steel wires of 200 µm diameter and 300 µm height (4 × 4 array), where the tips were cut tangentially to obtain sharp tips. These assembled BMn were used to pierce dermatomed human skin either manually or using an electrically driven applicator. Badran et al. utilized a novel BMn device called a Dermaroller^®^, with different stainless steel needle lengths (150, 500, and 1500 µm) protruding out from a cylindrical assembly containing 24 circular arrays of 8 needles each (192 needles in total) [17,38,39].

**Polymer:** Polymers used for microneedle production must be water-soluble, biocompatible, and mechanically robust enough for skin insertion [40]. The most common method for producing polymer-based BMn is the solvent casting technique. This process involves creating a mold of the microneedle design, pouring a polymer solution into the mold, allowing it to dry, and then removing the polymer BMn from the mold. Soluble or hydrogel-based BMn are typically produced using this method, with polymers such as hydroxypropyl methylcellulose frequently employed [29]. Liu et al. reported that transdermal administration using polyethylene-glycol diacrylate combined with peptides shows excellent biocompatibility and can be readily produced through photo-polymerization, enabling precise structural control without reducing peptide activity. This delivery platform was evaluated in a keloid scar model, where it successfully suppressed the expression of collagen I, a key marker of keloid formation, thereby highlighting its potential therapeutic application [41,42]. To complement this, Table S1 summarizes the sterilization techniques available for biodegradable polymers, along with their compatibility, advantages, and implications for reducing disease transmission.

**Glass**: Glass BMn are typically hollow and are manufactured using wet etching or a micropipette puller. They possess sufficient strength for skin penetration and are biocompatible. Hollow and glass BMn enable accurate microinjection into the dermis, either through the insertion of a single needle using rotary drilling followed by partial retraction for infusion, or by employing a microneedle array driven by mechanical trembling. Wang et al. demonstrated this approach by inserting hollow type glass microneedles into hairless rodent skin in vivo and human cadaver skin in vitro. The technique was validated through imaging of infused dye molecules, insulin, polymeric microparticles, and cells within the skin using brightfield and fluorescence microscopy [43]. Additionally, glass is easy to clean and stable under high temperatures and pressure. However, glass is prone to breaking, and if the tip of a glass microneedle fractures and remains embedded in the skin, it could lead to irritation or granuloma formation [29].

**Ceramics:** Ceramic materials are gaining significant attention in the development of biodegradable microneedles (BMn) because of their excellent biocompatibility, high mechanical strength, and safe biodegradability within the body. Common ceramics used in BMn fabrication include alumina (Al_2_O_3_), calcium phosphate (CaP), and calcium sulfate (CaSO_4_). Alumina is particularly valued for its superior strength and chemical stability, making it ideal for applications requiring durability during skin penetration, while maintaining good biocompatibility for medical use. Calcium phosphate, which closely resembles the mineral composition of natural bone, offers both biocompatibility and controlled biodegradability, making it well-suited for transdermal drug delivery systems.

Similarly, calcium sulfate exhibits favorable mechanical properties and biodegradability, further supporting its potential in safe and effective microneedle applications. Several studies have examined the use of these ceramics in BMn fabrication. For instance, Liu et al. investigated alumina for its mechanical robustness and stability [44], while Weijiang et al. demonstrated that calcium sulfate combined with gelatin enables efficient transdermal insulin delivery through microneedles that safely dissolve in the body [45]. Collectively, these findings underscore the promise of ceramic-based materials in advancing BMn technologies for transdermal drug delivery and vaccine administration. Some of the advantages and disadvantages of the materials used for BMn are summarized in Table 1.

## 5. Techniques for BMn Fabrication

Various materials are used in the fabrication of BMn, each requiring specific manufacturing techniques. Metallic BMn are typically produced through methods such as laser cutting, laser ablation, wet drawing, metal electroplating, and micro-molding. Silicon BMn are generally created using wet etching, dry etching, and three-dimensional laser cutting. For ceramic BMn, common techniques include micro-molding and sintering lithography. The fabrication of polymer BMn often involves methods like micro-molding, drawing lithography, photolithography, and drop-cast air blowing [3]. These techniques allow for the precise production of BMn tailored for specific applications in drug delivery and other medical treatment.

**Cutting:** Slicing is an efficient method for producing uniform BMn devices using a cutting machine. The BMn designed in 2D is bent at a 90-degree angle to form a 3D BMn. Electropolishing can be used to clean the needle tips or smooth rough surfaces [59,60]. However, it has some limitations, including reduced cutting precision and suboptimal surface finish, particularly due to the material strength and cutter size. Additionally, the wear of the cutting blades over time can impact product quality. This technique is typically suited for materials with moderate hardness, such as silicon and metal [3].

**Etching:** Etching is one of the primary fabrication techniques for BMn, involving the removal of material through a chemical reaction between an etchant and the substrate. Etching can be categorized into wet etching and dry etching based on the type of etchant used. Wet etching uses liquid etchants to dissolve the substrate and shape it as desired. Although it has limitations, such as a narrow range of materials and lower selectivity, wet etching remains widely used in microfabrication due to its faster processing speed and the easier treatment of etchants compared to dry etching. In contrast, dry etching employs gases as etchants, offering strong directionality and a high-quality etching effect, which results in better resolution and potentially deeper etching. Physical techniques encompass ion milling and sputtering [29,61]. However, dry etching requires specialized equipment, higher industrial costs, and precise gas handling to prevent ion bombardment. Both wet and dry etching processes involve highly corrosive chemicals, posing risks of severe skin damage if proper safety measures are not followed. Therefore, etching operations demand skilled professionals. These techniques are primarily used for the fabrication of silicon and metal BMn [32].

**Photolithography:** Photolithography is a fabrication technique used to transfer a predefined design from a photomask onto a substrate coated with a light-sensitive material, typically using radiation sources such as ultraviolet (UV) light or X-rays. By precisely controlling the exposure and patterning parameters, this process enables the creation of three-dimensional microstructures on the substrate. The method begins with a refined photomask composed of a transparent base and a patterned protective layer. During UV exposure, variations in light intensity across the mask dictate the degree of exposure in different regions, leading to the formation of a 3D microstructure. Beyond traditional photomasks, advanced methods such as switchable mobile laser sources and variable UV intensity systems have been developed to produce complex 3D patterns.

Kathuria et al. introduced a mask-free photolithography technique for fabricating BMn, where a Cr/Au-coated glass slide was isotropically etched using HF/HCl to form a micro-lens array. This array focused on UV light to achieve precisely shaped BMn tips with angles of 14.7° for shorter needles and 16.3° for longer ones. Similarly, Lim et al. fabricated hollow BMn using photolithography by placing a photomask with transparent circular patterns on a polyethylene terephthalate (PET) film coated with a photopolymer. Upon UV exposure, the exposed regions polymerized and adhered to the PET film, and subsequent development and drying caused the structures to form due to capillary action. While photolithography remains one of the most effective techniques for producing BMn with high precision and uniformity, it is primarily limited to photopolymer-based materials. The relatively low Young’s modulus of these materials, however, can restrict their mechanical performance and practical applications in certain biomedical contexts [4,5].

**Drawing Lithography:** Drawing lithography was first introduced by Lee et al. in 2010. Unlike traditional methods that rely on molds or masks, this technique involves drawing a molten polymer from a 2D substrate to directly form a 3D needle-like structure [12]. The cost of the entire process is significantly reduced, as it eliminates the need for UV light equipment. Additionally, drawing lithography overcomes the length and sharpness limitations of BMn compared to other commonly used techniques. These advantages enable large-scale BMn production in a short time, offering a solution for fabricating longer BMn [14]. However, drawing lithography has some limitations.

Xiang et al. noted that the technique is restricted by material formulation and stacking, making it challenging to produce thick, high-density, and small BMn. To address these challenges, a self-stacking drawing lithography method was proposed. In this approach, a SU-8 film was attached to a SU-8 coated wafer to create a self-stacking mold, which effectively separates the SU-8 source and controls the material volume. This method successfully prevents the merging of BMn tips during the drawing process. The improved drawing lithography method facilitated material reloading and prevented BMn from merging, thus enabling the production of dense and scalable BMn [21]. However, drawing lithography still relies on flat substrates, which limits its application in creating complex structures. To overcome this issue, Chen et al. introduced magnetorheological fluids into the drawing lithography process. By adding 1 μm diameter iron particles to the material as a magnetic guide, and applying an external magnetic field, they improved the sharpness and length of the BMn significant [32].

## 6. Application of BMn

With rapid advancements in fabrication technologies, BMn of varying sizes, shapes, and materials can now be produced with high precision. The choice of material and geometric parameters—such as shape, aspect ratio, tip radius, length, and base width—plays a critical role in determining the mechanical strength, biocompatibility, and overall performance of BMn. Clinically ideal BMn must combine excellent biocompatibility, sufficient mechanical robustness, and the capacity to incorporate and deliver therapeutic agents, including proteins, peptides, and vaccines. These properties are essential for ensuring efficient drug release and reliable skin sensing performance. Through careful design optimization, BMn with superior physical, chemical, and biological characteristics have been developed, expanding their potential in transdermal sensing, biofluid extraction for diagnostics, and transdermal drug delivery [36]. Owing to their minimally invasive nature, reduced pain, and high precision, BMn have gained considerable attention for a range of biomedical applications. Their most notable uses are in healthcare, particularly in drug delivery and biosensing, which are highlighted as the primary areas of focus below:

**Transdermal Drug Delivery:** BMn effectively overcome the stratum corneum barrier, enabling the efficient delivery of therapeutic agents such as insulin, vaccines, and analgesics. These microneedle systems can be engineered to target drug release at specific sites, making them particularly useful for localized treatments, including arthritis and skin cancer. They have also demonstrated great potential in vaccine administration for diseases such as influenza, hepatitis, and COVID-19, offering precise and controlled dosing with minimal discomfort. Quinn et al. reported the use of microneedle arrays for targeted drug delivery to melanoma lesions, enabling localized administration of chemotherapeutic agents while significantly reducing systemic side effects [62]. In addition to drug delivery, microneedles can be utilized to extract interstitial fluid for biomarker monitoring, supporting real-time diagnostic assessments of glucose, cholesterol, and other metabolites. Furthermore, they have been integrated into wearable biosensors for continuous monitoring, particularly in diabetes management, where they enable painless and accurate glucose tracking.

**Transdermal Sensing:** Clinically important analytes such as glucose, biomarkers, and ions are essential indicators for disease diagnosis and health monitoring. The growing demand for minimally invasive methods to assess these parameters in peripheral blood and interstitial fluid (ISF) has spurred the advancement of BMn-based sensing systems. Since ISF contains many of the same biochemical constituents as blood and reflects physiological changes associated with various diseases, it serves as a valuable medium for real-time health assessment. The concentration of analytes in ISF can thus provide reliable diagnostic information for detecting and monitoring medical conditions. Moreover, BMn offer notable benefits, including minimal pain, reduced skin irritation, and greater patient comfort compared to conventional needles, which enhances compliance and supports continuous health monitoring. To enable quantitative measurement of clinically relevant biomarkers, BMn are often integrated with a range of analytical and sensing technologies, facilitating accurate and efficient detection [36].

**Biofluid Extractions:** Biological fluids, such as blood and interstitial fluid (ISF), contain valuable substances that provide extensive physiological and biochemical information. Analyzing these fluids is critical for diagnosing various diseases and evaluating therapeutic effectiveness. Blood collection devices that are safe, easy to use, and efficient in extracting small volumes of bio fluids play a significant role in point-of-care (POC) diagnostics. However, conventional blood collection methods are often painful, can cause bleeding, and require highly trained professionals. BMn offer an ideal solution for transdermal bio fluid extraction due to their low cost, high safety, and ease of use. Among BMn, hollow BMn and solid BMn are primarily employed for blood extraction [28].

## 7. Disease Treatment

**Figure 5:**
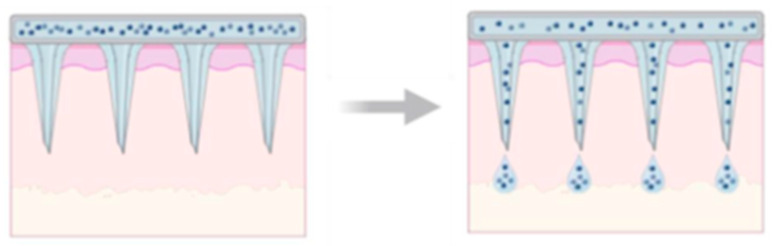
Schematic representation of microneedle-based transdermal drug delivery. (**Left**) Microneedles are loaded with a drug and applied to the skin, penetrating the outer epidermal layer. (**Right**) Upon application, the microneedles dissolve or release the encapsulated drug directly into the dermal tissue, enhancing drug absorption and therapeutic efficacy.

**Cargo/Vaccine delivery:** Since their introduction to drug delivery in 1998, BMn have seen remarkable advancements. Innovations in materials, fabrication techniques, and delivery strategies have established BMn as a promising alternative to traditional subcutaneous self-injections for disease management. This minimally invasive approach allows efficient transdermal delivery of therapeutic agents. Hollow BMn are particularly well-suited for transporting larger substances, including cells, as illustrated in Figure 5, while coated, swellable, or dissolving BMn are highly effective for delivering smaller molecules such as insulin [13].

BMn have emerged as a novel and effective platform for vaccine delivery, offering benefits such as minimal invasiveness, ease of administration, and enhanced immune responses. By targeting the dermal and epidermal layers rich in immune cells, BMn facilitate efficient antigen uptake by Langerhans cells and dendritic cells. Dissolving BMn encapsulate vaccines within their structure and release the antigen payload upon insertion into the skin. Coated BMn deliver vaccines pre-applied to their surfaces, allowing precise dosing and controlled release, while hollow BMn are suitable for administering liquid vaccines and larger payloads. For instance, Sullivan et al. demonstrated that dissolving BMn could effectively deliver influenza vaccines, eliciting immune responses comparable to traditional intramuscular injections [63]. Similarly, Matriano et al. reported that coated BMn could induce strong antibody titers for measles and rubella vaccines [64]. More recently, BMn have been explored for COVID-19 vaccination, with Kim et al. showing that dissolving BMn delivering spike protein antigens produced robust neutralizing antibody responses [65]. Beyond improving immunogenicity, BMn also simplify vaccine storage and distribution. Many systems can stabilize vaccines at room temperature, reducing reliance on cold-chain logistics—a major advantage for resource-limited regions. Although challenges such as limited payload capacity and large-scale manufacturing persist, ongoing advances in materials and fabrication techniques continue to enhance the practicality and effectiveness of BMn-based vaccine delivery.

**Microelectronic System Integrated BMn Devices for Sensing and Therapy:** With advancements in soft electronics, materials science, and fabrication techniques, microelectronic device-integrated BMn systems are becoming a reality, expanding the applications of BMn from traditional transdermal sensing and drug delivery to smart diagnostics and therapies. These integrated systems can convert biological signals into electrical signals, enabling comprehensive healthcare monitoring. For instance, hollow BMn sensors were modified with catechol-coated carbon paste to detect tissue tyrosinase levels, a biomarker associated with skin melanoma [61]. Upon contact with tyrosinase, catechol is oxidized to benzoquinone, producing a measurable amperometric signal. The wearable BMn sensor, coupled with a flexible electronic board, captures this signal and wirelessly transmits the data to a mobile device for analysis. Moreover, microelectronic systems in BMn can also convert various signals into therapeutic actions, offering innovative solutions for personalized medicine and remote health management. These advancements demonstrate the potential of BMn in bridging diagnostics with real-time therapeutic interventions, significantly enhancing the scope of minimally invasive healthcare technologies.

**Application of Microneedle in Psoriasis:** Psoriasis is a chronic, immune-mediated, polygenic inflammatory skin disorder that often relapses and affects approximately 2% of the global population, significantly reducing patients’ quality of life. Its exact cause remains unclear, despite extensive research efforts [29]. Key immune-related pathogenic factors, including TNF-alpha, IL-17A, and IL-23, have been widely recognized as central to its development. Common symptoms of psoriasis include erythema, scales, thickened skin, and red papules, often accompanied by itching, desquamation, and visible plagues, which profoundly affect patients’ daily lives. Unfortunately, there is currently no definitive cure for psoriasis [22]. Treatment options typically involve topical medications, physical therapies, and systemic drugs aimed at preventing disease progression. Topical treatments, while widely used, can be time-consuming, messy, and inconvenient due to their oily and sticky nature [26]. Physical therapies require strict adherence to treatment schedules, while systemic treatments are often associated with side effects, such as hepatotoxicity, renal impairment, and hypertension, particularly with drugs like methotrexate (MTX), cyclosporine A (CYA), and retinoic acid.

Additionally, oral and injectable biologics, despite their targeted approach and improved efficacy, are hindered by high costs and limited accessibility. Given these limitations, BMn are emerging as an innovative drug delivery platform for psoriasis treatment. BMn can offer painless, minimally invasive delivery of therapeutic agents directly into the affected skin layers, overcoming issues of poor penetration, reduced efficacy, and systemic side effects associated with conventional approaches. This novel method holds great promise in enhancing treatment outcomes, improving patient compliance, and potentially reducing the overall cost of psoriasis management [6].

**Ocular drug delivery:** The application of microneedle arrays has greatly advanced ocular drug delivery, particularly for macromolecules that are difficult to administer via traditional topical methods. Dissolving biodegradable microneedles (BMn) play a key role in enhancing the absorption of both small and large molecules, offering a promising strategy for treating ocular conditions. Age-related eye diseases, including diabetic retinopathy, posterior uveitis, and age-related macular degeneration, as well as conditions like glaucoma, can lead to significant vision impairment. Other ocular problems, such as fungal infections, further complicate treatment [2]. Conventional drug delivery approaches often fail to deliver sufficient drug concentrations to the target site. In response, BMn have emerged as a minimally invasive and highly efficient alternative, improving the transdermal delivery of drugs and genetic material. Among these, dissolving BMn is particularly advantageous, as they soften and dissolve upon penetrating sensitive ocular tissues, enabling controlled and effective drug release without causing significant discomfort or damage to the delicate ocular environment [21].

Studies have demonstrated that BMn enhances the bioavailability of therapeutic agents for ocular applications. For instance, Rojekar et al. (2024) highlighted the use of dissolving BMn for the sustained release of anti-VEGF drugs in age-related macular degeneration, showing improved therapeutic outcomes compared to conventional intravitreal injections [66]. Similarly, Zhang et al. (2023) [67] reported the successful delivery of antifungal agents using dissolving BMn in animal models, significantly reducing fungal load and improving recovery rates. These studies underline the potential of BMn to transform ocular therapeutics by ensuring precision, minimizing invasiveness, and enhancing patient compliance [67].

**Biomacromolecule Delivery:** The development of BMn has been driven by the need to understand and overcome the diffusion limitations of skin, particularly for biomacromolecules whose movement varies across different skin layers. Factors such as molecular weight and exposure time significantly influence the rate of diffusion [37]. By analyzing these barriers, strategies have been devised to enable targeted delivery of macromolecules through the epidermal and dermal layers, laying the groundwork for innovative skin-targeted clinical technologies. The skin serves as a critical route for administering bioactive molecules, offering both local and systemic therapeutic effects. BMn and transdermal patches provide the added advantage of bypassing hepatic first-pass metabolism, improving delivery efficiency. However, the stratum corneum remains the primary barrier, restricting both drug penetration and the detection of circulatory biomarkers. Many conventional topical and transdermal formulations are unable to effectively cross this barrier [14], highlighting the importance of microneedle technologies.

I. **Therapeutic Proteins:** Bovine serum albumin (BSA) is commonly used as a model protein to study transdermal delivery via BMn. Cheung et al. employed focused ion beam (FIB) techniques to fabricate BMn, enhancing BSA absorption using a “poke and patch” approach. [4].

II. **Insulin**: Insulin delivery has been extensively explored as an alternative to subcutaneous injections for diabetes management. Resnik et al. developed hollow silicone BMn capable of microinjecting insulin. In vivo studies showed effective infusion of fast-acting insulin, with BMn-based delivery producing a moderate glucose reduction but a significant increase (40–50%) in serum insulin levels, reflecting more efficient hormone administration [33].

III. **Vitamins:** Addressing vitamin deficiencies is a major public health concern. Kim et al. demonstrated the use of coated BMn loaded with PLGA nanoparticles for vitamin D delivery. Cholecalciferol-loaded nanoparticles were prepared via emulsion-solvent evaporation and coated onto solid metal BMn using a 5% (*w*/*v*) PVP solution. The optimized polymeric formulation achieved an 81.08% delivery efficiency, a five-fold improvement over conventional transdermal creams enhanced with chemical permeation agents, which delivered only 16.28% [3]. This body of research highlights the potential of BMn to efficiently deliver therapeutic proteins, hormones, and vitamins, overcoming traditional skin barriers and providing a minimally invasive alternative for systemic and localized treatment.

## 8. Delivery Systems of Microneedle Arrays

**Diffusion of Microneedle Arrays in Vaccine Delivery:** BMn fabricated from mechanically robust, water-soluble polymers are designed to penetrate the skin’s outermost layer, the stratum corneum, and rapidly dissolve within the viable epidermis and dermis. This enables direct delivery of vaccine components into the skin’s microenvironment, resulting in high local concentrations that allow for dose-sparing, reducing the amount of vaccine required, lowering costs, and minimizing potential toxicity [1]. Although the stability of each vaccine within BMn must be evaluated under different temperatures and storage conditions, studies indicate that BMn-embedded vaccines can remain stable for extended periods without reliance on costly cold-chain storage. Furthermore, preclinical and clinical studies have demonstrated that BMn offer a safe and well-tolerated platform for effective immunization strategies [3].

Currently, most vaccines are administered via intramuscular or subcutaneous injections. Currently, most vaccines are delivered via intramuscular or subcutaneous injections. However, skin offers a highly attractive alternative due to its rich population of antigen-presenting cells (APCs), including Langerhans cells in the viable epidermis and dendritic cells and macrophages in the dermis, which are critical for initiating robust immune responses. Unlike traditional needles that deposit vaccines into muscle tissue—an area with relatively low immunological activity—microneedle arrays precisely target these skin-resident APCs. For example, NanoPatch technology demonstrated that skin-targeted vaccination elicited a stronger immune response using only 1/100th of a standard intramuscular dose, while avoiding the pain and discomfort associated with conventional injections. BMn also overcome challenges related to the limited diffusion of macromolecules across the skin. The NanoPatch, a high-density microneedle array, enables efficient delivery across the dermal-epidermal junction, a barrier that normally restricts the passage of both hydrophilic and lipophilic molecules [22]. This barrier arises from the tight adhesion of stratum basale cells via tight junctions, desmosomes, and linker proteins, as well as the densely packed dermal collagen in the papillary layer, which impedes the movement of large molecules. In contrast, the looser collagen network in the deeper dermis offers less resistance to diffusion. By physically disrupting the stratum corneum and the dermal-epidermal junction, BMn facilitate efficient delivery of macromolecules directly to target immune cells within the skin [33].

Understanding and quantifying the diffusion of macromolecules within the skin is essential for designing advanced microscale devices that enable efficient transdermal drug delivery. Research has examined the movement of biomolecules across all layers of ex vivo human skin using minimally invasive microprojection arrays to deposit macromolecules, followed by imaging with non-invasive multiphoton microscopy (MPM). These studies revealed that smaller molecules (~70 kDa) are cleared rapidly from the skin within 30 min, whereas larger molecules (~2000 kDa) show significant retention. Diffusion rates generally increase from the skin surface (~3–8 μm^2^/s) to the papillary dermis (~1–20 μm^2^/s, depending on the model), but a marked reduction occurs at the dermal-epidermal junction (~0.7–3 μm^2^/s), which limits macromolecule passage into the dermis unless the barrier is directly disrupted by microprojections [33]. The human skin models used in these studies are thought to closely mimic in vivo conditions. Integrating minimally invasive clinical delivery tools with non-invasive imaging techniques can provide critical insights into macromolecule diffusion in individual patients, guiding the development of personalized nanoscale delivery systems. Quantifying diffusion across different molecular weights and skin layers represents a key advancement in the precise design of microneedle geometries, drug formulations, and fabrication methods for optimized transdermal therapies [27].

**Microneedle Array Systems for Long-Acting Drug Delivery:** The prevalence of chronic diseases, such as type 2 diabetes and cardiovascular conditions, is expected to increase significantly in the coming years. These conditions often require long-term medication regimens, which can disrupt patients’ daily lives and substantially raise healthcare costs [10]. Conventional immediate-release drug delivery systems face multiple challenges, including poor patient compliance, missed doses, fluctuating plasma drug levels, and the heightened risk of toxicity due to frequent dosing. These factors collectively reduce treatment efficacy, adherence, and overall cost-effectiveness. Long-acting drug delivery systems, based on sustained and controlled release mechanisms, offer a solution by maintaining consistent plasma drug concentrations over extended periods. These systems can improve patient compliance compared to daily medications or injections and enable site-specific drug delivery, concentrating on the therapeutic agent where it is most needed while minimizing systemic toxicity [37].

Typically fabricated from biodegradable and durable materials, long-acting drug delivery systems can be tailored to release drugs over hours, weeks, or even months. They address key issues associated with traditional drug delivery methods and are particularly advantageous for patients who struggle with adherence, such as those with psychiatric disorders. Polymeric micro- or nanoparticle-based injectable systems represent another advanced approach for sustained release. Microspheres and nanoparticles are the primary engineered particles used for long-lasting injectables [27]. These particles can be categorized as polymeric micro/nanospheres or micro/nanocapsules. Polymeric microspheres consist of a particle matrix, while nanocapsules feature an inner matrix (often oily) enclosed by a polymer shell. Both types are produced using a variety of biodegradable synthetic polymers, such as polylactic acid (PLA) and poly(lactide-co-glycolide) acid (PLGA) and are available in a wide range of sizes. These carriers have been extensively utilized for drug delivery across diverse anatomical locations, demonstrating significant versatility and efficacy [19].

**Drug Delivery via Porous BMn Technologies:** BMn systems offer a promising alternative to traditional hypodermic and subcutaneous needles by avoiding direct nerve contact while maintaining sufficient mechanical strength to penetrate the skin without bending or breaking, thereby improving patient compliance. These BMn often contain randomly distributed pores that facilitate both drug delivery and interstitial fluid sampling. While polymer-based porous BMn may have limited mechanical strength compared to solid microneedles, they provide advantages such as biocompatibility, ease of fabrication, and structural stability [37]. The mechanism of drug delivery via porous BMn differs from that of solid microneedles, though there are some common features. The mechanism of drug delivery via porous BMn differs from solid microneedles, while sharing some similarities. Porous BMn arrays are typically designed as single-unit delivery systems, where the entire microneedle array, including the backplate, acts as a reservoir for liquid or dry drug formulations. For liquid drugs, the formulation is loaded into the pores of the BMn array and diffuses into the dermal layers upon insertion. As the microneedle pores are depleted, additional drug can diffuse from the backplate reservoir through the BMn into the skin, combining diffusion-based delivery with microneedle-assisted pretreatment, while the BMn remain embedded throughout the process [36].

For dry formulations, the drug is loaded into the pores and dried using heat, vacuum, or freeze-drying. Upon insertion, interstitial fluid (ISF) enters the pores through capillary action, hydrating and dissolving the dry drug, which then diffuses into the skin. This approach enables controlled and efficient delivery by harnessing the skin’s natural ISF [27]. By integrating mechanical precision with effective drug release, porous BMn systems represent a significant advancement in transdermal therapeutic technologies, offering minimally invasive, reliable, and versatile drug administration.

## 9. Discussion and Conclusions

BMn represents a significant breakthrough in transdermal drug delivery, providing a minimally invasive, patient-compliant, and efficient alternative to traditional drug administration routes. Their diverse structures, such as solid, hollow, dissolving, and hydrogel-forming microneedles, allow for controlled, targeted, and painless drug delivery, minimizing systemic side effects and improving therapeutic efficacy. The integration of BMn with point-of-care and diagnostic platforms further highlights their potential in advancing personalized medicine, vaccination, and disease monitoring.

Despite the significant promise of biodegradable microneedles (BMn) for drug delivery, several limitations must be addressed to fully realize their potential. The primary challenge is their limited drug-loading capacity due to their small size, which constrains their use for delivering large doses or supporting sustained therapy. This limitation can be partially addressed by applying multiple patches simultaneously or replacing patches periodically during treatment. Another critical barrier is the poor solubility of certain drugs, as effective BMn delivery requires sufficient solubility; advanced solubilization strategies are therefore necessary to expand the range of compatible therapeutics [3]. Additionally, most BMn are designed for rapid drug dissolution, restricting their use to single-dose applications. Developing sustained-release BMn capable of continuous drug delivery is essential, particularly for chronic conditions requiring long-term therapy [2]. Fabrication challenges also remain, as producing master molds with high precision and reproducibility—often via deep reactive ion etching—is both costly and technically demanding, limiting large-scale production to specialized facilities [4]. Regulatory hurdles further complicate commercialization, as approval is typically granted for each specific application rather than the BMn platform itself, slowing market entry and innovation. Streamlining regulatory pathways could significantly enhance adoption and accelerate the broader application of BMn technologies [40].

## 10. Limitations and Future Directions of BMn

Future perspectives for BMn will involve leveraging their versatility to address global health challenges. For example, BMn’s potential in diabetes management is particularly promising, with adhesive polymer patches designed to deliver insulin effectively while enhancing therapeutic outcomes [22]. Furthermore, BMn’s integration with nanoparticles holds immense potential for improving drug solubility, reducing toxicity, and increasing efficacy, expanding their application in diagnostics and targeted therapies such as breast cancer treatment [37]. With advancements in material science, sustained drug release technologies, cost-effective fabrication methods, and streamlined regulatory processes, several researchers have investigated the use of MNPs loaded with adriamycin for the treatment of cutaneous squamous cell carcinoma (SCC) skin cancer, evaluating both their safety and therapeutic effectiveness, and this method has already completed Phase I testing, with overall trial completion anticipated by the end of 2024 (ClinicalTrials.gov identifier: NCT05377905). Another advantage of BMs is that they penetrate deeper without touching the nerves, especially in children (aged 7 to 18 years old). Three blood collection methods are commonly employed to determine blood sugar levels, namely, microneedle blood collection and two other widely accepted technically complex methods (i.e., intravenous (IV) catheters and Lancet). Through the above measures, future research will determine whether microneedle patch technology should be the first choice for monitoring blood glucose levels in diabetic children (ClinicalTrials.gov: NCT02682056). BMn is poised to revolutionize drug delivery systems, offering pain-free, efficient, and patient-compliant solutions for a variety of medical conditions.

## Figures and Tables

**Table 1:** An overview of the advantages and disadvantages, fabrication techniques, and manufacturing limitations of BMn.

Materials	Fabrication Technique	Applications	Advantages	Disadvantage	Limitation	References
Silicon	Deep reactive Etching, Lithography	Drug delivery	Excellent mechanical properties and versatile	high manufacturing costs	limited flexibility, high immunogenicity, low biocompatibility and biodegradability, less clinical application	[46,47,48]
Metal	Laser cutting, laser ablation, Template synthesis, Lithographic patterning	Gene and drug delivery	Excellent mechanical properties and superior tensile strength.	Susceptibility to fractures, corrosion, and limited biocompatibility	Corrosion susceptibility, Mechanical Fragility, Pain Perception, Cost concerns	[49,50,51,52]
Polymer	Micromolding, Hydrogel-creating, Dissolving, Hydrogel-forming, Coated	Insulin delivery	Excellent biocompatibility, complete biodegradability, and zero waste post-use.	Lower Mechanical Strength, Manufacturing Complexity, Swelling and Deformation	Skin elasticity complicates insertion, causing the BMn to separate quickly.	[53,54,55,56]
Glass	Micromolding, solvent casting	Drug delivery	Good biocompatibility	Mechanical properties are more difficult to achieve, stability problems, storage issues.	High cost, intricate and lengthy fabrication, multistep processing, brittle nature liable to break within skin	[57,58]
Ceramics	Template synthesis	Insulin delivery	High hardness, Thermal stability, High compressive strength, Versatility	Difficult to machine, Low tensile strength, Brittleness, Limited conductivity	During synthesis, insulin’s biological activity decreased from 95% at 37 °C to 25% at higher temperatures.	[45]

## Data Availability

Data is available within the article or from the corresponding author upon reasonable request.

## Supplementary Materials

The following supporting information can be downloaded at https://www.mdpi.com/article/10.3390/mps8060134/s1: Table S1: Summarizing sterilization techniques for biodegradable polymers, their compatibility, advantages and impact on disease transmission. References [68,69,70,71] is cited in the Supplementary Materials.

## References

[1] Xie L., Zeng H., Sun J., Qian W. (2020). Engineering Microneedles for Therapy and Diagnosis: A Survey. Micromachines.

[2] Henry S., McAllister D.V., Allen M.G., Prausnitz M.R. (1999). Microfabricated microneedles: A novel approach to transdermal drug delivery. J. Pharm. Sci..

[3] Jung J.H., Jin S.G. (2021). Microneedle for transdermal drug delivery: Current trends and fabrication. J. Pharm. Investig..

[4] Gupta J., Gill H.S., Andrews S.N., Prausnitz M.R. (2011). Kinetics of skin resealing after insertion of microneedles in human subjects. J. Control. Release Off. J. Control. Release Soc..

[5] Kim E., Erdos G., Huang S., Kenniston T.W., Balmert S.C., Carey C.D., Raj V.S., Epperly M.W., Klimstra W.B., Haagmans B.L. (2020). Microneedle array delivered recombinant coronavirus vaccines: Immunogenicity and rapid translational development. eBioMedicine.

[6] Prausnitz M.R. (2004). Microneedles for transdermal drug delivery. Adv. Drug Deliv. Rev..

[7] Dang N., Liu T.Y., Prow T.W. (2017). Nano- and Microtechnology in Skin Delivery of Vaccines. Micro and Nanotechnology in Vaccine Development.

[8] Chen Z., Lin Y., Lee W., Ren L., Liu B., Liang L., Wang Z., Jiang L. (2018). Additive Manufacturing of Honeybee-Inspired Microneedle for Easy Skin Insertion and Difficult Removal. ACS Appl. Mater. Interfaces.

[9] Johnson A.R., Procopio A.T. (2019). Low cost additive manufacturing of microneedle masters. 3D Print. Med..

[10] Kearney M.C., Caffarel-Salvador E., Fallows S.J., McCarthy H.O., Donnelly R.F. (2016). Microneedle-mediated delivery of donepezil: Potential for improved treatment options in Alzheimer’s disease. Eur. J. Pharm. Biopharm. Off. J. Arbeitsgemeinschaft Fur Pharm. Verfahrenstechnik e.V..

[11] Andranilla R.K., Anjani Q.K., Hartrianti P., Donnelly R.F., Ramadon D. (2023). Fabrication of dissolving microneedles for transdermal delivery of protein and peptide drugs: Polymer materials and solvent casting micromoulding method. Pharm. Dev. Technol..

[12] Donnelly R.F., Majithiya R., Singh T.R., Morrow D.I., Garland M.J., Demir Y.K., Migalska K., Ryan E., Gillen D., Scott C.J. (2011). Design, optimization and characterisation of polymeric microneedle arrays prepared by a novel laser-based micromoulding technique. Pharm. Res..

[13] van der Maaden K., Luttge R., Vos P.J., Bouwstra J., Kersten G., Ploemen I. (2015). Microneedle-based drug and vaccine delivery via nanoporous microneedle arrays. Drug Deliv. Transl. Res..

[14] Vora L.K., Moffatt K., Tekko I.A., Paredes A.J., Volpe-Zanutto F., Mishra D., Peng K., Raj Singh Thakur R., Donnelly R.F. (2021). Microneedle array systems for long-acting drug delivery. Eur. J. Pharm. Biopharm. Off. J. Arbeitsgemeinschaft Fur Pharm. Verfahrenstechnik e.V..

[15] Nguyen T.T., Oh Y., Kim Y., Shin Y., Baek S.K., Park J.H. (2021). Progress in microneedle array patch (MAP) for vaccine delivery. Human Vaccines Immunother..

[16] Lutton R.E., Larrañeta E., Kearney M.C., Boyd P., Woolfson A.D., Donnelly R.F. (2015). A novel scalable manufacturing process for the production of hydrogel-forming microneedle arrays. Int. J. Pharm..

[17] Verbaan F.J., Bal S.M., van den Berg D.J., Groenink W.H., Verpoorten H., Lüttge R., Bouwstra J.A. (2007). Assembled microneedle arrays enhance the transport of compounds varying over a large range of molecular weight across human dermatomed skin. J. Control. Release Off. J. Control. Release Soc..

[18] Bystrova S., Luttge R. (2011). Micromolding for ceramic microneedle arrays. Microelectron. Eng..

[19] Cai B., Xia W., Bredenberg S., Engqvist H. (2014). Self-setting bioceramic microscopic protrusions for transdermal drug delivery. J. Mater. Chem. B.

[20] Ramadon D., McCrudden M.T.C., Courtenay A.J., Donnelly R.F. (2022). Enhancement strategies for transdermal drug delivery systems: Current trends and applications. Drug Deliv. Transl. Res..

[21] Khan S., Hasan A., Attar F., Babadaei M.M.N., Zeinabad H.A., Salehi M., Alizadeh M., Hassan M., Derakhshankhah H., Hamblin M.R. (2021). Diagnostic and drug release systems based on microneedle arrays in breast cancer therapy. J. Control. Release Off. J. Control. Release Soc..

[22] Guillot A.J., Cordeiro A.S., Donnelly R.F., Montesinos M.C., Garrigues T.M., Melero A. (2020). Microneedle-Based Delivery: An Overview of Current Applications and Trends. Pharmaceutics.

[23] Lee H.S., Ryu H.R., Roh J.Y., Park J.H. (2017). Bleomycin-Coated Microneedles for Treatment of Warts. Pharm. Res..

[24] Tekko I.A., Permana A.D., Vora L., Hatahet T., McCarthy H.O., Donnelly R.F. (2020). Localised and sustained intradermal delivery of methotrexate using nanocrystal-loaded microneedle arrays: Potential for enhanced treatment of psoriasis. Eur. J. Pharm. Sci..

[25] Donnelly R.F., McCrudden M.T., Zaid Alkilani A., Larrañeta E., McAlister E., Courtenay A.J., Kearney M.C., Singh T.R., McCarthy H.O., Kett V.L. (2014). Hydrogel-forming microneedles prepared from “super swelling” polymers combined with lyophilised wafers for transdermal drug delivery. PLoS ONE.

[26] Gao Y., Hou M., Yang R., Zhang L., Xu Z., Kang Y., Xue P. (2019). Highly Porous Silk Fibroin Scaffold Packed in PEGDA/Sucrose Microneedles for Controllable Transdermal Drug Delivery. Biomacromolecules.

[27] Hu X., Zhang H., Wang Z., Shiu C.Y.A., Gu Z. (2021). Microneedle Array Patches Integrated with Nanoparticles for Therapy and Diagnosis. Small Struct..

[28] Niu L., Chu L.Y., Burton S.A., Hansen K.J., Panyam J. (2019). Intradermal delivery of vaccine nanoparticles using hollow microneedle array generates enhanced and balanced immune response. J. Control. Release Off. J. Control. Release Soc..

[29] Indermun S., Luttge R., Choonara Y.E., Kumar P., du Toit L.C., Modi G., Pillay V. (2014). Current advances in the fabrication of microneedles for transdermal delivery. J. Control. Release Off. J. Control. Release Soc..

[30] Burgeson R.E., Christiano A.M. (1997). The dermal-epidermal junction. Curr. Opin. Cell Biol..

[31] Ko M.S., Marinkovich M.P. (2010). Role of dermal-epidermal basement membrane zone in skin, cancer, and developmental disorders. Dermatol. Clin..

[32] Wei J.C.J., Haridass I.N., Crichton M.L., Mohammed Y.H., Meliga S.C., Sanchez W.Y., Grice J.E., Benson H.A.E., Roberts M.S., Kendall M.A.F. (2018). Space- and time-resolved investigation on diffusion kinetics of human skin following macromolecule delivery by microneedle arrays. Sci. Rep..

[33] Banga A.K. (2009). Microporation applications for enhancing drug delivery. Expert Opin. Drug Deliv..

[34] McGrath L.M., Pennington B.F., Shanahan M.A., Santerre-Lemmon L.E., Barnard H.D., Willcutt E.G., Defries J.C., Olson R.K. (2011). A multiple deficit model of reading disability and attention-deficit/hyperactivity disorder: Searching for shared cognitive deficits. J. Child Psychol. Psychiatry Allied Discip..

[35] Thakur R.R., Tekko I.A., Al-Shammari F., Ali A.A., McCarthy H., Donnelly R.F. (2016). Rapidly dissolving polymeric microneedles for minimally invasive intraocular drug delivery. Drug Deliv. Transl. Res..

[36] Ullah A., Kim C.M., Kim G.M. (2018). Porous polymer coatings on metal microneedles for enhanced drug delivery. R. Soc. Open Sci..

[37] Donnelly R.F., Raj Singh T.R., Woolfson A.D. (2010). Microneedle-based drug delivery systems: Microfabrication, drug delivery, and safety. Drug Deliv..

[38] Badran M.M., Kuntsche J., Fahr A. (2009). Skin penetration enhancement by a microneedle device (Dermaroller) in vitro: Dependency on needle size and applied formulation. Eur. J. Pharm. Sci. Off. J. Eur. Fed. Pharm. Sci..

[39] Zhao Z., Chen Y., Shi Y. (2020). Microneedles: A potential strategy in transdermal delivery and application in the management of psoriasis. RSC Adv..

[40] Liu S., Yeo D.C., Wiraja C., Tey H.L., Mrksich M., Xu C. (2017). Peptide delivery with poly(ethylene glycol) diacrylate microneedles through swelling effect. Bioeng. Transl. Med..

[41] Hulimane Shivaswamy R., Binulal P., Benoy A., Lakshmiramanan K., Bhaskar N., Pandya H.J. (2025). Microneedles as a Promising Technology for Disease Monitoring and Drug Delivery: A Review. ACS Mater. Au.

[42] Terashima S., Tatsukawa C., Suzuki M., Takahashi T., Aoyagi S. (2019). Fabrication of microneedle using poly lactic acid sheets by thermal nanoimprint. Precis. Eng..

[43] An M., Liu H. (2017). Dissolving Microneedle Arrays for Transdermal Delivery of Amphiphilic Vaccines. Small.

[44] Yu W., Jiang G., Liu D., Li L., Tong Z., Yao J., Kong X. (2017). Transdermal delivery of insulin with bioceramic composite microneedles fabricated by gelatin and hydroxyapatite. Mater. Sci. Eng. C.

[45] Das A., Singha C., Bhattacharyya A. (2019). Development of silicon microneedle arrays with spontaneously generated micro-cavity ring for transdermal drug delivery. Microelectron. Eng..

[46] Pradeep Narayanan S., Raghavan S. (2019). Fabrication and characterization of gold-coated solid silicon microneedles with improved biocompatibility. Int. J. Adv. Manuf. Technol..

[47] Li Y., Zhang H., Yang R., Laffitte Y., Schmill U., Hu W., Kaddoura M., Blondeel E.J.M., Cui B. (2019). Fabrication of sharp silicon hollow microneedles by deep-reactive ion etching towards minimally invasive diagnostics. Microsyst. Nanoeng..

[48] Rajabi M., Roxhed N., Shafagh R.Z., Haraldson T., Fischer A.C., Wijngaart W.V.D., Stemme G., Niklaus F. (2016). Flexible and Stretchable Microneedle Patches with Integrated Rigid Stainless Steel Microneedles for Transdermal Biointerfacing. PLoS ONE.

[49] Li J., Liu B., Zhou Y., Chen Z., Jiang L., Yuan W., Liang L. (2017). Fabrication of a Ti porous microneedle array by metal injection molding for transdermal drug delivery. PLoS ONE.

[50] Mukaibo H., Johnson E.A., Mira F., Andrion K., Osteikoetxea X., Palma R., Martin C.R. (2015). Template-synthesized gold microneedle arrays for gene delivery to the Chlamydomonas reinhardtii chloroplast. Mater. Lett..

[51] Senel M., Dervisevic M., Voelcker N.H. (2019). Gold microneedles fabricated by casting of gold ink used for urea sensing. Mater. Lett..

[52] Nguyen H.X., Bozorg B.D., Kim Y., Wieber A., Birk G., Lubda D., Banga A.K. (2018). Poly (vinyl alcohol) microneedles: Fabrication, characterization, and application for transdermal drug delivery of doxorubicin. Eur. J. Pharm. Biopharm. Off. J. Arbeitsgemeinschaft Fur Pharm. Verfahrenstechnik e.V..

[53] Kim J.Y., Han M.R., Kim Y.H., Shin S.W., Nam S.Y., Park J.H. (2016). Tip-loaded dissolving microneedles for transdermal delivery of donepezil hydrochloride for treatment of Alzheimer’s disease. Eur. J. Pharm. Biopharm. Off. J. Arbeitsgemeinschaft Fur Pharm. Verfahrenstechnik e.V..

[54] Park Y.-H., Ha S.K., Choi I., Kim K.S., Park J., Choi N., Kim B., Sung J.H. (2016). Fabrication of degradable carboxymethyl cellulose (CMC) microneedle with laser writing and replica molding process for enhancement of transdermal drug delivery. Biotechnol. Bioprocess. Eng..

[55] Nguyen H.X., Banga A.K. (2018). Delivery of Methotrexate and Characterization of Skin Treated by Fabricated PLGA Microneedles and Fractional Ablative Laser. Pharm. Res..

[56] Norman J.J., Choi S.-O., Tong N.T., Aiyar A.R., Patel S.R., Prausnitz M.R., Allen M.G. (2013). Hollow microneedles for intradermal injection fabricated by sacrificial micromolding and selective electrodeposition. Biomed. Microdevices.

[57] Gupta J., Park S.S., Bondy B., Felner E.I., Prausnitz M.R. (2011). Infusion pressure and pain during microneedle injection into skin of human subjects. Biomaterials.

[58] Gill H.S., Prausnitz M.R. (2007). Coated microneedles for transdermal delivery. J. Control. Release Off. J. Control. Release Soc..

[59] Shakya A.K., Ingrole R.S.J., Joshi G., Uddin M.J., Anvari S., Davis C.M., Gill H.S. (2019). Microneedles coated with peanut allergen enable desensitization of peanut sensitized mice. J. Control. Release Off. J. Control. Release Soc..

[60] Kim Y.C., Park J.H., Prausnitz M.R. (2012). Microneedles for drug and vaccine delivery. Adv. Drug Deliv. Rev..

[61] Srivannavit O., Gulari M., Gulari E., LeProust E., Pellois J.P., Gao X., Zhou X. (2004). Design and fabrication of microwell array chips for a solution-based, photogenerated acid-catalyzed parallel oligonuclotide DNA synthesis. Sens. Actuators A Phys..

[62] Quinn H.L., Larrañeta E., Donnelly R.F. (2016). Dissolving microneedles: Safety Considerations and Future Perspectives. Ther. Deliv..

[63] Sullivan S.P., Koutsonanos D.G., Del Pilar Martin M., Lee J.W., Zarnitsyn V., Choi S.O., Murthy N., Compans R.W., Skountzou I., Prausnitz M.R. (2010). Dissolving polymer microneedle patches for influenza vaccination. Nat. Med..

[64] Matriano J.A., Cormier M., Johnson J., Young W.A., Buttery M., Nyam K., Daddona P.E. (2002). Macroflux^®^ Microprojection Array Patch Technology: A New and Efficient Approach for Intracutaneous Immunization. Pharm. Res..

[65] Xiaojie S., Yu L., Lei Y., Guang Y., Min Q. (2020). Neutralizing antibodies targeting SARS-CoV-2 spike protein. Stem Cell Res..

[66] Rojekar S., Parit S., Gholap A., Manchare A., Nangare S., Hatvate N., Sugandhi V., Paudel K., Ingle R. (2024). Revolutionizing Eye Care: Exploring the Potential of Microneedle Drug Delivery. Pharmaceutics.

[67] Zhang Q., Zhang Z., Zou X., Liu Z., Li Q., Zhou J., Gao S., Xu H., Guo J., Yan F. (2023). Nitric oxide-releasing poly(ionic liquid)-based microneedle for subcutaneous fungal infection treatment. Biomater. Sci..

[68] Smith F., Sabri A.H., Heppel M., Fonseca I., Chowdhury F., Cheung K., Willmor S., Rawson F., Marlow M. (2022). The clinical and translational prospects of microneedle devices, with a focus on insulin therapy for diabetes mellitus as a case study. Int. J. Pharm..

[69] Umeyor C.E., Shelke V., Pol A., Kolekar P., Jadhav S., Tiwari N., Anure A., Nayak A., Bairagi G., Agale A. (2023). Biomimetic microneedles: Exploring the recent advances on a microfabricated system for precision delivery of drugs, peptides, and proteins. Futur. J. Pharm. Sci..

[70] Dai Z., Ronholm J., Tian Y., Sethi B., Cao X. (2016). Sterilization techniques for biodegradable scaffolds in tissue engineering applications. J. Tissue Eng..

[71] González García L.E., MacGregor M.N., Visalakshan R.M., Ninan N., Cavallaro A.A., Trinidad A.D., Zhao Y., Hayball A.J.D., Vasilev K. (2019). Self-sterilizing antibacterial silver-loaded microneedles. Chem. Commun..

