# Supplementary material for: Biodegradable Microneedle for Enhanced Transdermal Drug Delivery: Trends and Techniques
Source: Methods Protoc. 2025 Nov 4;8(6):134. doi: 10.3390/mps8060134 (PMC12641650; doi:10.3390/mps8060134)
Supplement: Supplementary file 1 [file mps-08-00134-s001.zip › mps-3856289-supplementary.pdf]

Table S1: Summarizing sterilization techniques for biodegradable polymers, their compatibility, advantages and impact on disease transmission.

Sterilization Method	Compatibility with BMn	Advantages	Limitations	Impact on Disease Transmission	References
Gamma irradiation	Suitable for many polymeric dissolving microneedles (e.g., PVP K90, hyaluronic acid), hydrogel-forming BMn	High penetration, low heat load, can sterilize packaged devices; no chemical residues	Can degrade or alter mechanical properties, drug release profiles; some polymers are more sensitive. Needs correct dose	Very effective against microbes including sterilizing pathogens, reduces disease transmission if MNs are used single-use and properly sterilized.	68,69
Ethylene Oxide (EtO)	Used for materials sensitive to heat or wet steam; many biodegradable polymers.	Low temperature sterilization, good for heat-/moisture-sensitive drugs; penetrates packaging well.	Requires long aeration to remove toxic residues; may affect chemical integrity of some polymer-drug.	If properly executed, reduces pathogen load; residual EtO or by-products if not removed may pose safety concerns.	70
Autoclave	Limited compatibility; feasible for metal, glass or certain robust polymers. For many biodegradable polymer BMn, it is not suitable.	Cheap, fast, widely available; well-understood technology.	High heat and moisture can deform, dissolve, or weaken MN structure; may destroy drug or affect release kinetics.	When usable, can sterilize effectively; but if polymer integrity compromised, risk of incomplete sterilization or loss of function.	69
Dry Heat	Some heat-resistant polymers can tolerate; many biodegradable systems cannot.	Simple method; no moisture; no chemical residue.	Requires high temperature (e.g., ~160 °C) for long durations, which often degrade biodegradable polymers or affect drug.	Can effectively kill microorganisms, but damage to MN could lead to failures or microdamage that affects safety.	69
Self-sterilizing / Antimicrobial Coatings	Compatible with dissolving BMn; e.g., silver-loaded MNs (e.g. nano-silver with carboxymethylcellulose)	Prevents microbial growth at the insertion site; may allow safer handling; potentially reduces need for harsh sterilization.	Coating may alter dissolution, possible toxicity or irritation; stability of the antimicrobial agent; cost issues.	Helps reduce risk of infection during use; may complement other sterilization methods; but alone might not assure total sterility.	71

References

68. Smith, F.; Sabri, A. H.; Heppel, M.; Fonseca, I.; Chowdhury, F.; Cheung, K.; Willmor, S.; Rawson, F.; Marlow, M. The clinical and translational prospects of microneedle devices, with a focus on insulin therapy for diabetes mellitus as a case study. *Int J Pharm* **2022,** *628*, 122234.
69. Umeyor, C. E.; Shelke, V.; Pol, A.; Kolekar, P.; Jadhav, S.; Tiwari, N.; Anure, A.; Nayak, A.; Bairagi, G.; Agale, A.; Raut, V.; Bahadure, S.; Chaudhari, A.; Patravale, V. B. Biomimetic microneedles: exploring the recent advances on a microfabricated system for precision delivery of drugs, peptides, and proteins. *Futur J Pharm Sci.* **2023,** *9* (1), 103.
70. Dai, Z.; Ronholm, J.; Tian, Y.; Sethi, B.; Cao, X. Sterilization techniques for biodegradable scaffolds in tissue engineering applications. *J. Tissue Eng.* **2016,** *7*, 2041731416648810.
71. González García, L. E.; MacGregor, M. N.; Visalakshan, R. M.; Ninan, N.; Cavallaro, A. A.; Trinidad, A. D.; Zhao, Y.; Hayball, A. J. D.; Vasilev, K., Self-sterilizing antibacterial silver-loaded microneedles. *Chem. Commun.* **2019,** *55* (2), 171–174.
